# Polychlorinated Biphenyls Induce Cytotoxicity and Inflammation in an In Vitro Model of an Ocular Barrier

**DOI:** 10.3390/ijms26030916

**Published:** 2025-01-22

**Authors:** Alessia Cosentino, Aleksandra Agafonova, Luca Cavallaro, Rosaria Ester Musumeci, Chiara Prinzi, Cinzia Lombardo, Maria Teresa Cambria, Carmelina Daniela Anfuso, Gabriella Lupo

**Affiliations:** 1 Department of Biomedical and Biotechnological Sciences, School of Medicine, University of Catania, 95123 Catania, Italy; alessia1993@hotmail.it (A.C.); aleksandraaagafonova@gmail.com (A.A.); chiara27chiara@gmail.com (C.P.); cinzialombardo@hotmail.com (C.L.); gabriella.lupo@unict.it (G.L.); 2 Department of Civil Engineering and Architecture, University of Catania, 95123 Catania, Italy; luca.cavallaro@unict.it (L.C.); rosaria.musumeci@unict.it (R.E.M.)

**Keywords:** polychlorinated biphenyls, Aroclor 1254, phospholipase A_2_, mitogen-activated protein (MAP) kinase, prostaglandins E2, interleukin 1β (IL-1β), tumor necrosis factor α, transforming growth factor β, nuclear factor NF-kB

## Abstract

Polychlorinated biphenyls (PCBs) are heterogeneous, synthetic, and widespread organochlorine compounds, and are one of the persistent organic pollutants present in improperly dumped waste and electronic equipment (e-waste), with a high bioaccumulation potential. In this study, the toxicity of Aroclor 1254 (a mixture of commercial PCBs) in human corneal epithelial cells (HCEpiCs), in an in vitro model of an ocular barrier, was evaluated. Aroclor 1254 (0.1–10 μg/mL) reduced cell viability, trans-endothelial electric resistance (TEER) and cell migration. Moreover, it induced an inflammatory response, as indicated by the increase in cPLA_2_ activity, PGE2 production, phosphorylation of ERK 1/2 and p-38, and release of inflammatory cytokines. Aroclor 1254 can damage corneal cells, compromising the integrity of the eye’s outermost barrier. This damage may facilitate the occurrence of infectious processes that are physiologically prevented by the corneal barrier.

## 1. Introduction

Polychlorinated biphenyls (PCBs) are a class of 209 chemical compounds consisting of biphenyls with benzene rings that bind a variable number of chlorine substitutions at different positions. From a chemical point of view, PCBs are classified into dioxin-like or non-dioxin-like congeners, with different affinities for the aryl hydrocarbon receptor [1].

PCBs had been widely used for about 50 years as industrial additives because of their particular characteristics, such as electrical insulation and heat resistance, until their production was banned in the late 1970s [2]. Despite their replacement with less toxic materials, since more than 1 million tons of PCBs have been produced, it has been estimated that one-quarter of the PCB waste produced is present in the environment [3]. PCBs are characterized by a high bioaccumulation potential, as they are stable and resistant to environmental degradation. For this reason, they persist both in the environment and in living organisms [4]. High emission of PCBs has been found in large industrial areas due to the mechanical dismantling and recycling of electronic waste (e-waste), such as hardware and transformers of obsolete electrical equipment [5]. Such increased levels in the environment, associated with serious health risks for frontline workers, are a cause for great concern.

For PCBs, the occupational exposure limit, which is known as the threshold limit value or TLV, is 0.5 mg/m^3^. TLVs represent the concentrations of airborne chemicals in the environment, below which, as demonstrated by scientific research [6], the majority of workers can be exposed daily for all their working life without experiencing any toxic health effects. The TLV values relating to various chemical substances, and their biological exposure indices (BEIs), are published every year by the American Association of Industrial Hygienists (ACGIH), and in Italy, after authorization from the ACGIH itself, by the Italian Association of Industrial Hygienists (AIDII). However, it is necessary to specify that, despite their importance, TLVs do not represent the limit beyond which damage to health is certain to occur for any individual. In fact, individual susceptibility to one or more chemical substances differs based on age, sex, predisposing genetic factors, and pre-existing pathologies. Furthermore, the type of work performed—heavy or light—can influence individuals’ susceptibility to chemicals and possible biological responses. Therefore, TLVs represent a recommendation index for risk prevention in the workplace [7].

The toxicity of PCBs has now drawn worldwide attention. Exposure to PCBs has been shown to be associated with liver dysfunction, endocrine dysfunction, reduced bone mineral density and cancer. Moreover, their ability to induce neurotoxicity and an increased risk of dementia and Parkinson’s disease have also been demonstrated [8]. PCBs are mainly generated by waste incineration and industrial, domestic combustion of coal and wood, as well as the improper dumping of e-waste, which is mainly composed of polycyclic aromatic hydrocarbons and particulates [8]. PCBs can be transported long distances, and are released into the environment. Humans come into contact them through the air, especially in densely populated industrial areas. PCBs present in landfills can be released into rivers, lakes, and soil, where they accumulate, exposing the population to serious risks, particularly when they affect the external corneal barrier. Not to be underestimated, in addition to constant daily exposure, is a lack of eye contact among personnel working on ships during naval maneuvering inside ports, which can also lead to accidents that could expose workers to PCBs. Another category of at-risk workers is farmers or plant product operators, who come into contact with PCBs while harvesting or preparing for large-scale distribution.

Aroclor 1254 is a mixture of commercial PCBs that is commonly used in toxicology research. It contains approximately 60 PCB congeners, which include the ones that are mainly found in environmental samples, such as PCB47, PCB153, and PCB77 [9].

To date, there are no data on the effects of PCBs on the eye, which has a natural external defensive barrier, the cornea, that protects the internal tissues of the eye from any type of insult coming from the external environment. The cornea is a tissue that is transparent to light, with a structural function. Its transparency, permitted by collagen fibers arranged in orderly layers so as not to interfere with the passage of light radiation through it, allows light to be focused on the retina. To maintain transparency, the cornea is avascular [10]. On the surface, the cornea is covered by the epithelium, made up of multiple layers of continuously multiplying cells that form a real dynamic barrier capable of rapidly repairing an insult from an external cause. The innermost layer of the cornea is made up of perennial endothelial cells, which are never renewed. The endothelium regulates the water content of the cornea, which, in turn, affects the transparency of the cornea itself [11].

The cornea is the outermost anterior element of the eye, and therefore plays an important role in protecting the eye from environmental insults. The barrier function of the epithelium allows the underlying stroma to be protected from physical trauma, pathogens, and chemical substances. Following an insult, the cornea can lose its transparency, due to the infiltration of immune cells and the activation of keratocytes [12]. In most healthy individuals, a corneal epithelial wound heals quickly and effectively, usually within a couple of days, restoring both the cornea’s structure and function to the pre-injury state [13].

Air pollution has been shown to induce toxicity in corneal cells by causing an increase in the release of pro-inflammatory cytokines [14]. In the damaged cornea, the release of interleukin 1β (IL-1β) and tumor necrosis factor α (TNF-α), and an increased activity of cytosolic phospholipases A_2_ (cPLA_2_), have been demonstrated [15].

cPLA_2_ catalyzes the hydrolysis of the sn-2 position of membrane glycerophospholipids, mostly phosphatidylcholine, to release arachidonic acid (AA), a precursor of eicosanoids including prostaglandins E2 (PGE2) and leukotrienes (LTs) [16].

The involvement of PLA_2_ in response to contact with PCBs was demonstrated almost thirty years ago on neutrophils obtained from the peritoneal cavities of Sprague Dawley rats and treated with Aroclor 1242 [17].

In the following years, numerous studies were conducted that demonstrated the activation of PLA_2_, with the consequent release of prostaglandins, following contact with PCBs. Among these are those conducted on human platelets [18], cerebellar granule neurons [19], and liver epithelial cells [20].

Furthermore, it has been demonstrated that there exists a correlation between the activation of PLA_2_ and the triggering of the ERK 1/2 and p38 mitogen-activated protein (MAP) kinase signaling pathways in human extravillous cytotrophoblast (EVCT)-derived transformed cells exposed to PCBs [21].

Some evidence has suggested that the release of inflammatory cytokines, such as IL-1β, TNF-α, and transforming growth factor-β (TGF-β) transforming growth factor β (TGF-β), might play a key role in the effects of PCBs on different tissues [22].

The secretion of inflammatory cytokines in human endothelial cells treated with PCB-126 is regulated via nuclear factor NF-kB signaling [23]. Consequently, these molecules, upon release, may contribute to the host defense mechanism.

The production and storage of abundant e-waste has become a global environmental problem [24]. To the best of our knowledge, this is the first investigation into the mechanism by which PCBs can damage the corneal barrier.

The findings of this study could contribute to exploring the underlying mechanism involved in the inhibitory potential of Aroclor on the viability of corneal epithelial cells, which form the outermost barrier of the eye, and, consequently, on their ability to protect and heal wounds. The results are significant because they will evaluate the ocular toxicity of PCBs and could identify control strategies for workers’ health.

## 2. Results

### 2.1. HCEpiC Viability

**Figure 1:**
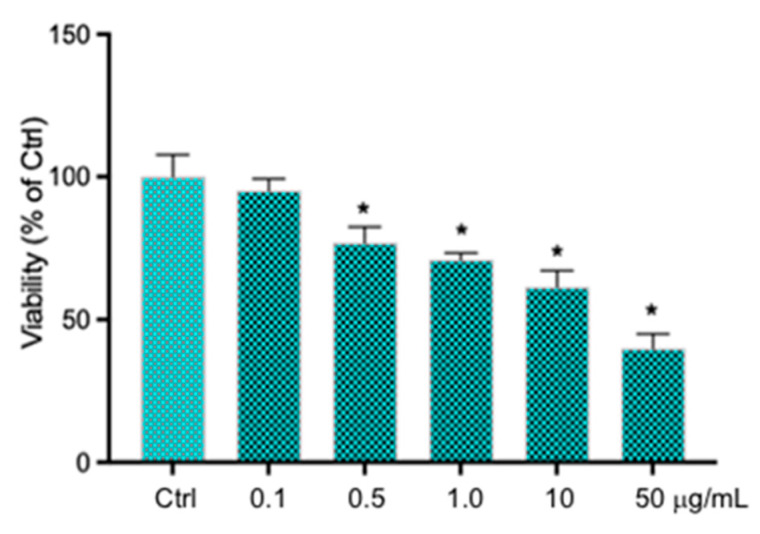
Cell viability, evaluated by MTT assays on HCEpiCs (human corneal epithelial cells) treated for 24 h with Aroclor 0.1–0.5–1–10–50 and 100 μg/mL. Values are expressed as mean ± SD of three independent experiments performed in triplicate. * *p* < 0.05 vs. untreated cells (CTRL). One-way ANOVA with Tukey–Kramer post hoc test.

In order to evaluate the effects of Aroclor on cell viability, MTT assays, after treating HCEpiCs with increasing concentrations of Aroclor (Aroclor 0.1–0.5–1–10–50 μg/mL), were performed. As shown in Figure 1, the concentration of 0.1 μg/mL caused no effect on cell viability, while Aroclor concentrations of 0.5–1–10 μg/mL induced a decrease in cell viability by about 24%, 30%, and 39%, respectively. A strong toxic effect was exerted by Aroclor at concentrations of 50 μg/mL, inducing a reduction in HCEpiC viability by 61%. Based on the results obtained, subsequent treatments were carried out with Aroclor at concentrations of 0.1, 1, and 10 μg/mL, excluding the higher concentration of 50 μg/mL, which is excessively toxic for HCEpiCs.

### 2.2. Transendothelial Electrical Resistance (TEER)

**Figure 2:**
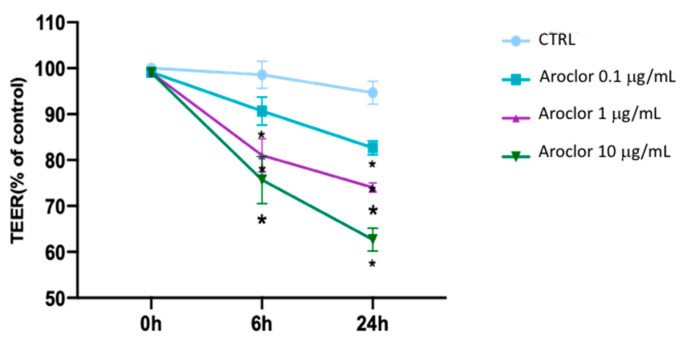
Assessment of barrier integrity in HCEpiC cultures by TEER measurements after treatment with Aroclor 0.1, 1, or 10 μg/mL, at times 0 (0 h), 6 h, and 24 h. Untreated HCEpiCs (human corneal epithelial cells) are indicated as CTRL. Values are means ± standard deviation (SD) of three independent experiments, each time in triplicate (*n* = 3). One-way ANOVA with Tukey–Kramer post hoc test. * *p* < 0.05 vs. 0 h.

The effects of Aroclor on corneal epithelial barrier integrity were evaluated by TEER assays. HCEpiCs were plated on Transwell membranes and incubated for 6 h and 24 h with different concentrations of Aroclor. As shown in Figure 2, Aroclor at 0.1 μg/mL did not induce significant changes in TEER after 6 h of incubation, while after 24 h, the TEER values were significantly reduced by 18%, compared with untreated HCEpiC (CTRL). Higher concentrations of Aroclor 1 μg/mL and 10 μg/mL significantly reduced the TEER by approximately 19% and 25% after 6 h, and by 26% and 38% after 24 h, respectively, compared with control cells.

### 2.3. HCEpiC Migration

**Figure 3:**
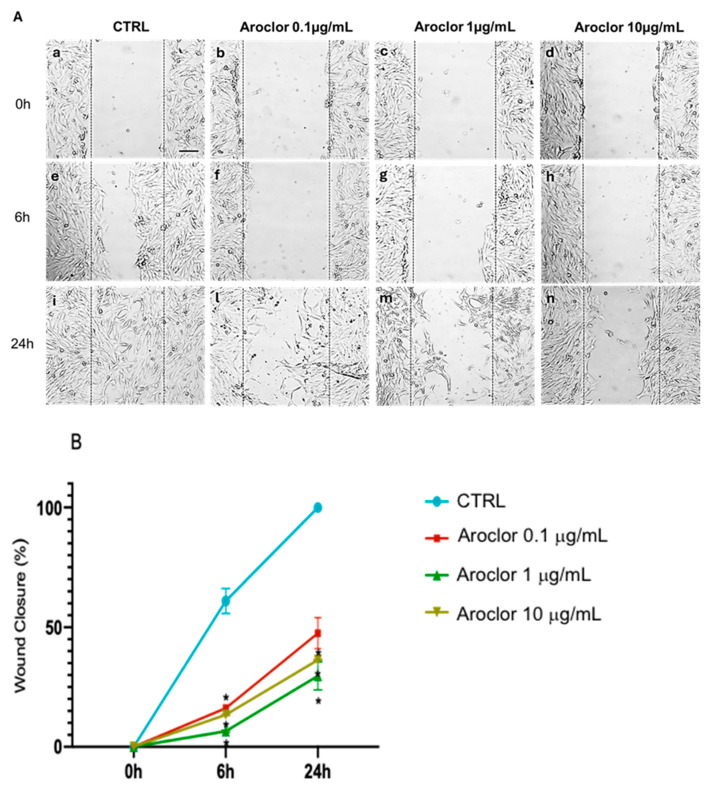
Effects of different concentrations of Aroclor on the migration of HCEpiCs (human corneal epithelial cells). (**A**): representative images of wound healing assays performed at 0 h, 6 h, and 24 h in the absence (control cells (CTRL), panels a, e, i, respectively) or in the presence of Aroclor with a concentration of 0.1 µg/mL (panels b, f, l), 1 µg/mL (panels c, g, m), or 10 µg/mL (panels d, h, n). All images were acquired with a Leica microscope using 20X magnification; scale bar = 200 μm. (**B**): relative quantification of wound closure percentage, carried out in the absence (CTRL) or in the presence of Aroclor 0.1 µg/mL, 1 µg/mL, or 10 µg/mL. The data are shown as the mean ± SD of *n* = 3 independent experiments. * *p* < 0.05 vs. CTRL. One-way ANOVA, followed by the Tukey–Kramer post hoc test.

A wound healing assay was used to study the impact of the treatment with Aroclor on the HCEpiCs’ capability of wound repair. In Figure 3A, at time zero, the width of the wound did not differ between the control and Aroclor treatments at all the concentrations used (panels a–d). At 6 h, the migration of cells in the control began to close the wound (panel e), unlike the cells treated with all three concentrations of Aroclor, where no migration occurred (panels f–h). After 24 h of incubation, the control cells almost completely closed the wound (panel i), and the cells treated with the lowest concentration of Aroclor (0.1 μg/mL) underwent a slow migration without being able to close the wound (panel l). Aroclor at concentrations of 1 μg/mL and 10 μg/mL was able to reduce the migration of HCEpiCs (panels m and n, respectively). In panel B, the percentage of wound closure is reported. After 24 h, wound closure was reduced by 53%, 64%, and 71% by Aroclor 0.1 µg/mL, 1 µg/mL, and 10 µg/mL, respectively, compared with the untreated control cells.

### 2.4. cPLA_2_ Protein Expression

**Figure 4:**
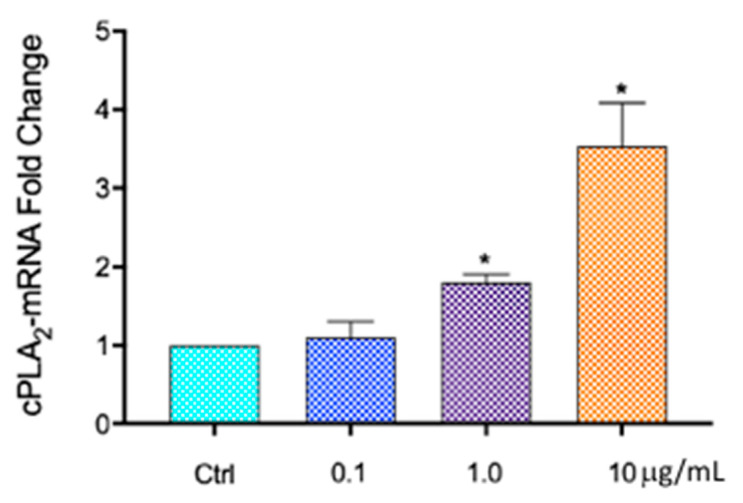
Effect of Aroclor on mRNA levels of cPLA_2_ in HCEpiCs (human corneal epithelial cells). Cells were treated for 24 h with Aroclor 0.1, 1, or 10 µg/mL. Each bar represents mean ± SD of three independent experiments, each in triplicate (*n* = 3). * *p* < 0.05 vs. CTRL. One-way ANOVA with Tukey–Kramer post hoc test. *p* < 0.05 vs untreated control cells (CTRL).

Because it has been demonstrated that cPLA_2_ activation, and the subsequent release of arachidonic acid from neutrophils exposed to PCBs, play a key role in the mechanism of PCB toxicity [17], we tested the mRNA levels of cPLA_2_ after treatment with different concentrations of Aroclor. In Figure 4, the results of quantitative RT-qPCR show a significant increase of about 1.8- and 3.5-fold in the mRNA levels of cPLA_2_ in HCEpiCs treated with Aroclor 1 and 10 µg/mL, respectively, compared with control cells. No significant differences were observed after treatment with the lower concentration of Aroclor, 0.1 µg/mL.

These data indicate that Aroclor can elicit its toxicity in HCEpiCs through an increase in the synthesis of cPLA_2_.

### 2.5. cPLA_2_ Activity and Prostaglandins E2 Release

To confirm the role of cPLA_2_ in HCEpiCs treated with Aroclor, cPLA_2_ activity was measured (Table 1). In HCEpiCs treated for 24 h with Aroclor 1 or 10 μg/mL, the enzymatic activity was significantly enhanced (about 1.8-fold and 2.5-fold, respectively) compared with untreated control cells. No significative activation of the enzyme activity by the treatment of HCEpiCs with Aroclor 0.1 μg/mL was found. Such a marked increase in enzymatic activity after treatment with Aroclor 10 μg/mL confirms the data obtained with the RT-PCR analysis, highlighting both an increase in enzyme transcription and its activation. The treatment of HCEpiCs with lower concentrations of Aroclor caused the activation of the enzyme, which was also physiologically present in the control, but in an inactive form.

Because Aroclor induced an increase in cPLA_2_ activity, which releases arachidonic acid that, in turn, is the substrate of COX-2, we measured the release of PGE2 in HCEpiCs incubated with or without Aroclor 1 or 10 μg/mL. PGE2 release (Table 2) increased by about 1.5 and 2.5 fold, respectively, in comparison to untreated control cells. These data confirmed that Aroclor triggered the inflammatory process in HCEpiCs.

### 2.6. ERK and p38 Protein Expression

**Figure 5:**
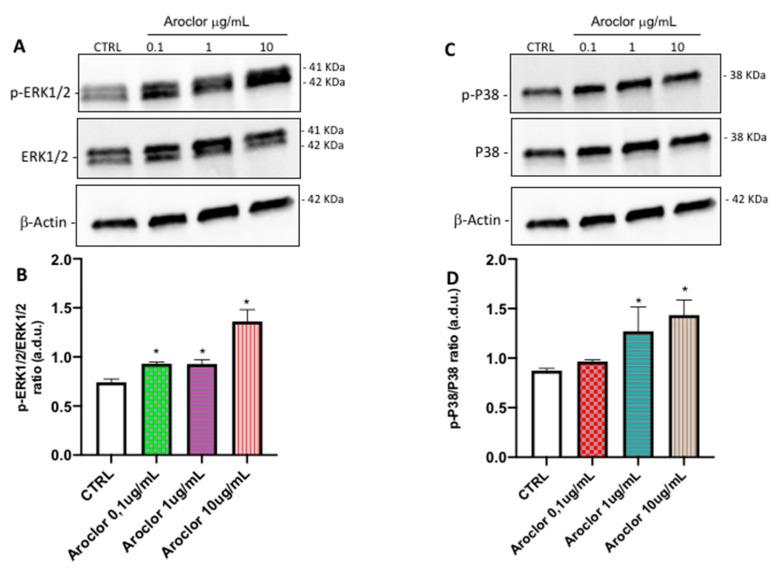
Activation of ERK 1/2 and p38 in HCEpiCs (Human corneal epithelial cells) incubated for 24 h in the absence (CTRL) or in the presence of Aroclor 0.1 µg/mL, 1 µg/mL, or 10 μg/mL. Immunoblot analysis of HCEpiC whole-cell lysates, using antibodies against phospho-ERK1/2 (p-ERK1/2) and total ERK1/2 (**A**), or phospho-p38 (p-P38) and total p38 (**C**). Blot was probed with anti β-actin antibody to verify equal loading of 30 μg proteins per lane. (**B**,**D**): Densitometric analyses of immunoblot, indicating protein quantification of each band (in arbitrary densitometry units, a.d.u.), carried out with Image J software (v1.52a, National Institutes of Health, Bethesda, MD, USA). Data are shown as mean ± SD of *n* = 3 independent experiments performed in triplicate. * *p* < 0.05 vs. CTRL. One-way ANOVA, followed by Tukey–Kramer post hoc test.

To explore the related signaling mechanisms, ERK1/2 and phospho-ERK1/2, p-38, and phospho-p38 MAPK levels were examined in HCEpiCs that were untreated or treated with Aroclor 0.1, 1, or 10 μg/mL, using Western blot analyses. As shown in Figure 5, panel A, the activated/phosphorylated form of ERK 1/2 is visible after treatment with increasing concentrations of Aroclor in comparison with the control cells. The p-ERK 1/2/ERK 1/2 ratio (graph in panel B) shows an increase of approximately 1.3-fold after treatment with Aroclor 0.1 and 1 μg/mL, and of approximately 1.5-fold after treatment with Aroclor 10 μg/mL, compared with untreated control cells.

In panel C, the phosphorylated form of p38 weakly increases after treatment with Aroclor in comparison with the control cells (panel C). The phospho-p38/p38 ratio (graph in panel D) shows an increase of approximately 1.5-fold and 1.8-fold after treatment with Aroclor 1 and 10 μg/mL, compared with untreated control cells, with no differences among the three concentrations used.

These data confirm, in corneal cells, the other results showing that PCB treatment activated the ERK1/2 and p38 MAPK signaling pathways in human extravillous cytotrophoblast-derived transformed cells [21].

### 2.7. Cytokine mRNA Transcription

**Figure 6:**
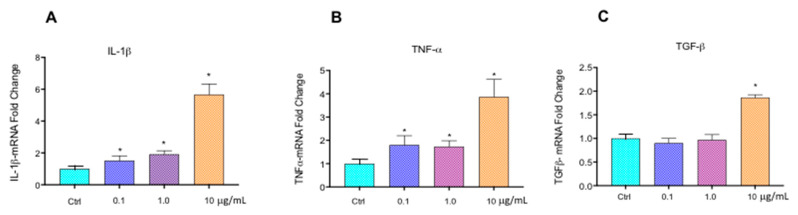
(**A**) Interleukin 1β (IL-1β), (**B**) tumor necrosis factor α (TNF-α), and (**C**) transforming growth factor β (TGF-β) mRNAs in HCEpiCs (Human corneal epithelial cells), untreated (CTRL) or treated for 24 h with Aroclor 0.1 µg/mL, 1 µg/mL, or 10 µg/mL. mRNA transcription was determined by reverse transcription-polymerase chain reaction (RT-PCR) analysis using respective primers described in Materials and Methods. Data are shown as mean ± SD of *n* = 3 independent experiments. * *p* < 0.05 vs. CTRL. Two-way ANOVA, followed by Tukey–Kramer post hoc test.

The effects of Aroclor on the induction of the transcription of inflammatory cytokines in HCEpiC cultures were determined by RT-PCR. The results shown in Figure 6, panel A, indicate that Aroclor significantly enhanced IL-1β mRNA by about 1.5–2- and 5.6-fold at the concentrations of 0.1–1 and 10 µg/mL, respectively, in comparison with the control cells. TNF-α mRNA increased by 1.8-, 1.7-, and 3.8-fold after treatment with 0.1, 1, and 10 μg/mL of Aroclor, respectively (panel B), and TGF-β mRNA increased by 1.8-fold only at the highest concentration of Aroclor, in comparison with the control cells (panel C). These results suggest that Aroclor could exert its effect by inducing the mRNA transcription of the cytokines that could play a synergistic effect in promoting inflammation.

### 2.8. NF-kB Protein Expression

**Figure 7:**
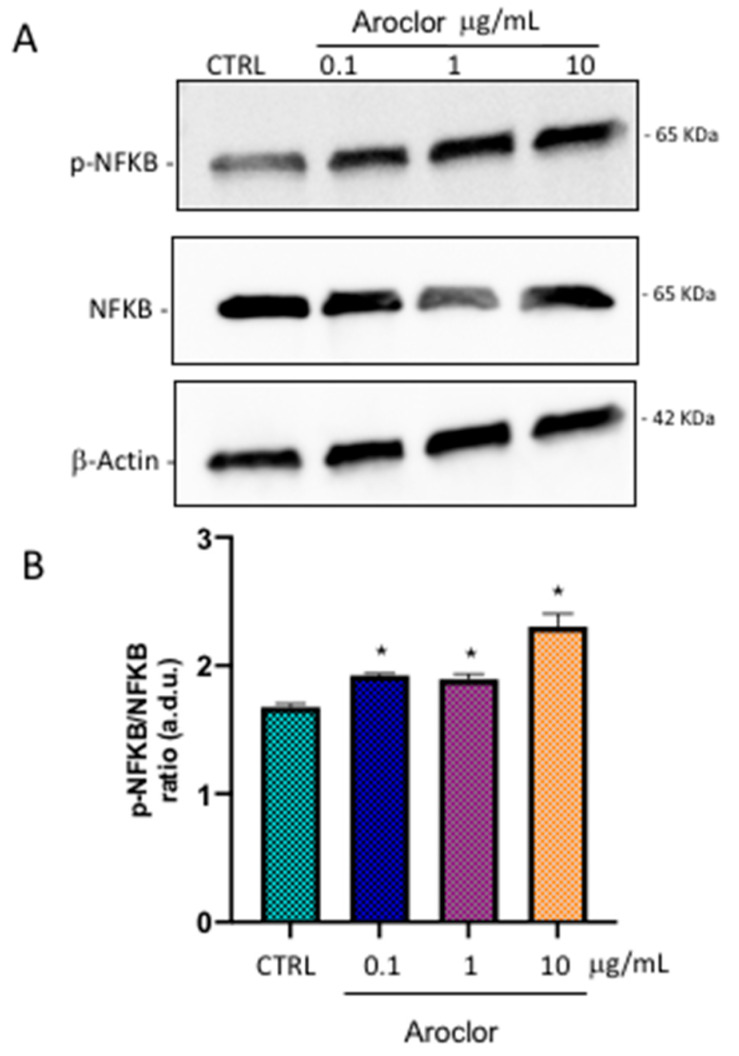
Western blot analysis of NF-kB and p-NF-kB. (**A**) Protein expression of NF-kB and p-NF-kB in HCEpiCs (Human corneal epithelial cells) untreated (CTRL) or treated for 24 h with Aroclor 0.1 μg/mL, 1 μg/mL, or 10 μg/mL. (**B**) Densitometric analysis of immunoblots, indicating protein quantification of each band (in arbitrary densitometry units, a.d.u.), carried out with Image J program. Quantitative analysis of results is expressed as p- NF-kB/NF-kB ratio. Data are shown as mean ± SD of *n* = 3 independent experiments performed in triplicate. * *p* < 0.05 vs. CTRL. One-way ANOVA, followed by Tukey–Kramer post hoc test.

Following exposure to different concentrations of Aroclor, HCEpiCs were evaluated for their protein expression of the total and the active (phosphorylated) form of NF-kB, using immunoblot analyses. In Figure 7, panel A, it can be seen that Aroclor induced an increase in the levels of active protein, in comparison with control cells. The total amount of NF-kB was very similar in the control cells and in the HCEpiCs treated with 0.1 µg/mL, 1 µg/mL, or 10 µg/mL of Aroclor. The quantitative analysis of immunoblot results, expressed as the p NF-kB/NF-kB ratio, is reported in panel B. The values indicate that Aroclor significantly increased the ratio by approximately 1.2-fold at the concentrations of 0.1 µg/mL and 1 µg/mL, and by 1.4-fold at the concentration of 10 μg/mL, in comparison with untreated cells (CTRL).

## 3. Discussion

The cornea plays an important role in refracting light onto the crystalline lens, which in turn focuses the light onto the retina. To perform this function, the corneal epithelium must continually renew itself to maintain a smooth optical surface and function as a barrier to protect the eye from various environmental insults. The adult corneal epithelium maintains its characteristic function thanks to an integrated process of cell proliferation and migration. Impairment of this process causes corneal alteration that can cause blindness. Research over the years has demonstrated that adequate coordination of cellular signaling pathways is responsible for corneal epithelial renewal and wound healing [25].

Over the years, PCBs have been linked to different pathologies that affect many organs [2]. To date, there are no studies on the effects of these substances on the eye’s external barrier, which protects it from external insults.

The results of our study demonstrate that treatment of HCEpiCs for 24 h with Aroclor, at all the concentrations used, had significant toxic effects on the cells that form the protective layer of our eyes.

The corneal epithelium is constantly exposed to chemical and biological insults from the external environment. In order to carry out its protective function, the cornea has a high TEER, determined by its cells being sealed together by tight junctions, and a high regenerative capacity, which leads it, when subjected to an insult, to activate the proliferation and migration of its cells [26]. We demonstrated that Aroclor reduced TEER values in a concentration-dependent manner, and is therefore capable of altering the protective barrier of our eyes. Moreover, in the wound healing process, Aroclor suppressed HCEpiC migration, highlighting that, when in contact with the eyes, it is capable of delaying the healing of a wound.

PCBs can cause inflammation of the vascular endothelium, leading to atherosclerotic events [27]. In our model, Aroclor induced an inflammatory response, both by activating cPLA_2_-mRNA transcription and by increasing its enzymatic activity, causing an accumulation of arachidonic acid (AA). These events were confirmed by an increase in the production of PGE2, the final product of the metabolism of AA by COX enzymes. Therefore, the activation of cPLA_2_ and the subsequent PGE2 release from HCEpiCs exposed to PCBs may be important in mechanisms of PCB toxicity.

It has been demonstrated that during corneal wound healing, ERK1/2 is activated, and plays an important role in the initiation of cell migration and proliferation [28]. In addition to ERK 1/2, the p38 signaling pathways play a role in promoting tissue repair by stimulating the migration of corneal cells during the healing process of a corneal injury, with cross-talk between these two MAP kinase signal cascades [29]. Moreover, it has also been shown that activation of the ERK1/2 signaling pathway is involved in the disruption of epithelial tight junctions and, therefore, in the barrier function [30].

Our results pointed out that the treatment of HCEpiC with Aroclor increased the activation/phosphorylation of ERK 1/2 and p38, confirming that the MAP kinase signaling pathway is upregulated in corneal cells subjected to an insult by Aroclor. The role played by the ERK signaling pathway in damaged corneal cells could represent a central point in Aroclor-induced toxicity, because the ERK pathway has been identified as a mediator of the corneal epithelial wound healing process in pharmacological intervention studies. In these studies, different models were used—rat models and human cells in vitro, stimulated with various compounds, such as diadenosine polyphosphates [31], sericin [32], and diquafosol [33]. Other studies on possible pharmacological interventions, which aim to activate the healing of a corneal wound and the restoration of barrier functions, have shown that p38 inhibition improves the proliferation of human corneal cells [34] and reduces cellular senescence [35]. Mao et al. have also recently demonstrated that the downregulation of the p38 signaling pathway stimulates the self-renewal of limbal stem cells, demonstrating that small molecules modulating the p38 MAPK signaling pathway ameliorate tissue-engineered corneal epithelium [36].

To date, there are no studies on the role of ERK and p38 in corneal cells treated with PCBs or, in particular, with Aroclor.

An important role in the orchestration of these processes is played by the release of pro-inflammatory cytokines. In particular, it has been shown that p38 MAPK activation depends on endogenous TGF-β in corneal wound healing, suggesting its participation in corneal tissue repair [37,38]. Moreover, Zhi-Yuan Li et al. demonstrated that TGF-β-driven NF-kB activation contributes to corneal epithelial senescence, characterized by changes in the corneal epithelium and associated with major eye diseases, including the highly prevalent dry eye disease [39]. Our results demonstrated that Aroclor induced an increase in inflammatory cytokine transcription in HCEpiC cultures. In particular, the most significant increase was found in IL-1β mRNA expression compared with that of TNF-α mRNA and TGF-β mRNA, which significantly increased after Aroclor treatment, but showed lower levels. Our present findings are in agreement with the literature, showing that increased circulating inflammatory cytokines are not only seen in various blinding eye diseases, but are also implicated in their pathogenesis [40].

The production of pro-inflammatory cytokines is closely correlated with the activation of NF-kB [41]. The obtained results demonstrated that, after the treatment of HCEpiCs with Aroclor, NF-kB protein expression increased in a concentration-dependent manner, confirming its correlation with inflammatory cytokine release. Indeed, it has been demonstrated that NF-kB, which responds to different types of extracellular insults, is activated to regenerate the damaged corneal epithelium. It plays its role in corneal cells by regulating the synthesis of retinoic acid, which induces the differentiation of corneal cells and their maintenance in physiological conditions [42]. Inflammation is a protective response that has the function of eliminating offensive agents, having the potential to damage different areas of our body. However, depending on the type of agent, the inflammatory response could be dysregulated and cause damage [43].

When the cells forming the outer layer of the cornea come into contact with Aroclor, they lose their barrier characteristics, due to the triggering of an inflammatory process that makes them unable to proliferate and heal the damage suffered. The signaling pathway activated involves cPLA_2_ and MAP kinases, with the subsequent release of prostaglandins and inflammatory cytokines.

## 4. Materials and Methods

All reagents and antibodies were purchased from Sigma (St. Louis, MO, USA) or Merck (Darmstadt, Germany), unless otherwise indicated.

### 4.1. Methods

#### Cell Cultures and Treatments

Human corneal epithelial cells (HCEpiC, Innoprot, P10871, Derio, Spain) were cultured in Corneal Epithelial Cell Complete Medium (Innoprot, P60131) at 37 °C, in a humidified atmosphere of 5% CO_2_. The culture medium was exchanged every other day, and the cultures were maintained until sub-confluence. For all experiments, cells were incubated in serum-free medium for 24 h with different concentrations of Aroclor.

Aroclor 1254 (Sigma, 11097-69-1) was dissolved in dimethyl sulfoxide (DMSO) to create a stock solution. Aliquots of stock solution were added to the incubation medium to yield the desired final concentrations of 0.1–10 μg/mL, with a final DMSO concentration of 0.1% (*v*/*v*). DMSO (1 μL/mL) had no significant effect either on the human corneal epithelial cells’ viability or TEER.

### 4.2. Cell Viability Assay

HCEpiC viability, after treatment with Aroclor, was determined by 3-[4,5-dimethylthiazol-2-yl]-2,5-diphenyl tetrasodium bromide (MTT assay, Chemicon, Temecula, CA, USA), as previously described [44,45]. Cells were seeded in 96-well plates at a density of 2 × 10^4^ cells per well, and incubated in medium overnight at 37 °C before treatments were given. Subsequently, different concentrations of Aroclor (0.1–0.5–1–10–50–100 μg/mL) were added to each well, except the wells with control solution (medium without Aroclor), for 24 h. After incubation, 10 μL MTT reagent (5 mg/mL) was added to each well, and the plates were incubated for 3 h at 37 °C. The formazan crystals were extracted with 100 μL DMSO. The absorbance was measured at 570 nm with a plate reader (Biotek Instruments, Elx-800). The obtained values were reported in graphs as percentages of the control.

### 4.3. Transendothelial Electrical Resistance Measurement (TEER)

HCEpiCs were plated in transwells (Costar, 3412, Washington, DC, USA) with a 0.4 μm pore size at a density of 7 × 10^4^ cells/cm^2^, and treated with Aroclor 0.1, 1, or 10 μg/mL for 6 h and 24 h. TEER was measured with the Millicell-ERS system (MERS 000 01; Millipore, AG, Volketswil, Switzerland), as previously described [46]. Blank values on empty transwells were recorded. The experimental values were expressed as ω × cm^2^, and were calculated by the following formula: [average resistance of experimental wells − average resistance of blank wells] × 0.33 (the area of the transwell membrane). In order to achieve optimal HCEpiC confluent monolayers, the TEER values were monitored for 3–4 days before the treatments with Aroclor.

### 4.4. Wound Healing Assay

The putative involvement of the treatment with Aroclor on corneal epithelial migration was evaluated in HCEpiCs by a standard wound healing assay [47]. Briefly, confluent cell monolayers were wounded by scratching with a P200 pipette tip. After washing with PBS 1 X, cells were incubated with a medium containing Aroclor 0.1, 1, or 10 μg/mL (at time 0). The wound area was analyzed from six different wells for each treatment, performed in three independent cell cultures. Photographs at 20X magnification, using a bright-field Leica microscope, were acquired at each time point of 0, 6, and 24 h from randomly selected fields to monitor wound closure. Image J software (Broken Symmetry Software, Bethesda, MD, USA) was used to analyze HCEpiC wound closure by evaluating the migration of the cellular front over time into the cell-free wound area.

### 4.5. Total RNA Isolation and Real-Time Quantitative RT-PCR

After treatments of the HCEpiCs with Aroclor, qRT-PCRs, to determine mRNA levels of cPLA_2_, IL-1β, TGF-β1 and TNF-α, were performed as previously described [48].

First-strand cDNA was reversely transcribed in 20 μL reaction volume with 200 U of SuperScript III, 50 ng random hexamers, 1.25 mM dNTP, 10 mM dithiothreitol, 50 mM Tris–HCl with a pH of 8.3, 75 mM KCl, and 3 mM MgCl_2_ (Invitrogen Life Technologies, Waltham, MA, USA). The reaction, carried out at 42 °C for 50 min, was stopped at 70 °C for 10 min. The PCR reactions were performed in 10 μL final volumes containing 1 μL of cDNA, 8 pmol of each primer, 1.7 mM dNTP mix, and 1 U TAQman Gold DNA polymerase (Applied Biosystem, Waltham, MA, USA) in 1 X Buffer II. The specific primers used are listed in Table 3.

Amplifications were carried out in a 7300 Real Time PCR System (Applied Biosystems, Thermo Fisher Scientific, Waltham, MA, USA). Negative controls were included in each assay. The qRT-PCR data were analyzed by the comparative threshold cycle method (∆∆Ct). All the samples were run in triplicate and normalized by the expression of the endogenous 18S rRNA gene.

### 4.6. Phospholipase A_2_ Assay and PGE2 Release

HCEpiCs were incubated for 24 h with Aroclor 0.1, 1, or 10 μg/mL. Control cells were incubated in a medium without Aroclor. At the end of the incubations, the cells were scraped with a rubber policeman, lysed as previously described [49], and equal amounts of cell lysates were used for the determination. Lysates were incubated in a 96-well plate with the substrate arachidonoyl-thio-phosphatidylcholine (ATPC), using a cPLA_2_ assay kit (Cayman Chemical, Ann Arbor, MI, USA), following the manufacturer’s instructions. The results, expressed as pmol of ATPC hydrolyzed per minute and per milligram protein (pmol/min/mg), were from three different experiments performed in triplicate.

To determine PGE2 release by HCEpiCs treated for 24 h with Aroclor 0.1, 1, or 10 μg/mL, aliquots of culture medium were analyzed using ELISA kits (PGE2, Cayman Chemical, Ann Arbor, MI, USA), according to the manufacturer’s instructions. Control cells were incubated in a medium without Aroclor. The results were from three different experiments performed in triplicate.

### 4.7. Immunoblot Analyses

After treatments with Aroclor 0.1, 1, and 10 μg/mL, cellular monolayers were washed twice with PBS and harvested mechanically, using a cell scraper. After centrifugation at 180× *g* for 5 min at 25 °C, the pellets were lysed for 30 min in RIPA buffer (Calbiochem-Merck, 20188, Darmstadt, Germany). Western blot analysis was performed as previously reported [50]. Immunoblots were detected by using the Odyssey Imaging System (LI-COR Biosciences, Lincoln, NE, USA). The intensity of protein bands was quantitated by ImageJ Software (NIH, Bethesda, MD, USA).

### 4.8. Statistical Analysis

Each experiment was carried out three times, each time in triplicate (*n* = 3). The data are reported as the mean ± SD. The different groups/conditions were compared by one-way analysis of variance (ANOVA) and the Tukey–Kramer post hoc test; *p* values < 0.05 were considered statistically significant. The statistical analysis and graph design were carried out by means of GraphPad Prism 7.00 software (GraphPad Inc., San Diego, CA, USA).

## 5. Conclusions

**Figure 8:**
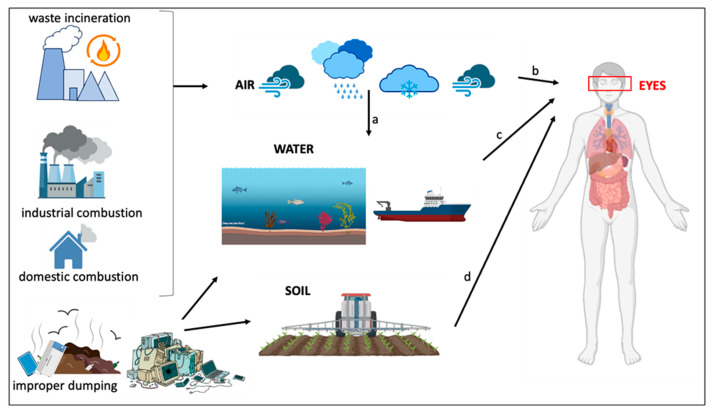
Human exposure to PCBs, derived from different sources, which can affect the eyes and, in particular, the external corneal barrier. (**a**) Atmospheric transport, which can contaminate water and soil; (**b**) contact with particulate matter in atmospheric air; (**c**) contact with contaminated water, even accidentally, by workers on ships, which can occur following errors in ship maneuvering within ports; (**d**) contact with contaminated soil by farmers or plant product operators, who come into contact with PCBs during preparation for large-scale distribution.

PCBs, generated by different sources, can contaminate water and soil (Figure 8). The accidental contact of workers with contaminated environments can affect the cornea’s outer layer cells, causing them to lose their ability to function as a barrier.

The observed reduction in cell viability, trans-endothelial electric resistance, and cell migration and the inflammatory response, indicated by the increase in cPLA_2_ activity, PGE2 production, ERK 1/2 and p-38 phosphorylation, and the release inflammatory cytokines, pointed to the impact of Aroclor 1254 on human corneal epithelial cells.

The findings highlight the mechanisms that regulate corneal cell response to Aroclor 1254, suggesting that targeting the activated signaling pathway could represent a potential pharmacological strategy to promote healing and corneal health.

## Figures and Tables

**Table 1:** cPLA_2_ activity in HCEpiCs (human corneal epithelial cells), incubated for 24 h in the absence (control) or in the presence of Aroclor 0.1, 1, or 10 µg/mL. All incubations were performed at 37 °C; PLA_2_ activity was measured following enzymatic hydrolysis of arachidonoyl thio-phosphatidylcholine (ATPC). Values (mean ± SD) are from three independent experiments, performed in triplicate (*n* = 3). * *p* < 0.05 vs. untreated control cells.

Treatment (μg/mL)	PLA_2_ Activity (pmol/min/mg)
Control	46.3 ± 5.6
Aroclor 0.1	49.8 ± 3.9
Aroclor 1.0	86.1 ± 6.2 *
Aroclor 10	115.1 ± 9.3 *

**Table 2:** Effect of Aroclor 0.1, 1, or 10 μg/mL on PGE2 production in HCEpiCs (Human corneal epithelial cells). ELISA kits (PGE2, Cayman Chemical, Ann Arbor, MI, USA) were used according to manufacturer’s instructions. Controls were performed by incubation of cells in medium without Aroclor. Values (mean ± SD) are from three independent experiments, performed in triplicate (*n* = 3). * *p* < 0.05 vs. untreated control cells.

Treatment (μg/mL)	PGE_2_ (pg/mL)
Control	354 ± 45
Aroclor 0.1	333 ± 58
Aroclor 1.0	535 ± 46 *
Aroclor 10	786 ± 61 *

**Table 3:** Primers used for RT-PCR.

Gene	Sequence (5′-3′)
TGF-β1	Fw CGTCTGCTGAGGCTCAAGT Rv CGCCAGGAATTGTTGCTGTA
cPLA_2_	Fw CTC TTG AAG TTT GCT CAT GCC CAG AC Rv GCA AAC ATC AGC TCT GAA ACG TCA GG
TNF-α	Fw AGC CCA TGT TGT AGC AAA CC Rv TGA GGT ACA GGC CCT CTG AT
IL-1β	Fw AGCTACGAATCTCCGACCAC Rv CGTTATCCCATGTGTCGAAGAA

## Data Availability

Data are available on request.
